# VALUECARE Model for Value-Based, Integrated Health and Social Care Services Delivery Supported by ICT for Older Adults

**DOI:** 10.5334/ijic.8931

**Published:** 2025-02-11

**Authors:** Mireia Ferri Sanz, Alejandro Gil-Salmerón, Maite Ferrando, Oscar Zanutto, E. L. S. Bally, Sara Ceron, Demi Cheng

**Affiliations:** 1 Senior Europa SL (Kveloce), Valencia, Spain; 2 International Foundation for Integrated Care, Oxford, UK; 3 International University of Valencia, Valencia, Spain; 4 ISRAA –Istituto per Servizi di Ricovero e Assistenza agli Anziani –Treviso (ISRAA), Italy; 5 Department of Public Health, Erasmus MC University Medical Center, Rotterdam, Netherlands

**Keywords:** co-design, care model, value-based health care, integrated care, older adults

## Abstract

**Introduction:**

Value-based healthcare is a current global trend in health and policy where digital technologies can play an important role to measure what matters to the people. The digitalisation of value-based healthcare is only possible if the care team and people trust in this new concept and the tools provided. In this framework, a participatory co-desing approach was implemented to identify the core elements of an innovative value-based, integrated health and social care delivery model supported by ICT solutions: the VALUECARE model for older people with comorbidities.

**Description:**

The main guiding principles for care delivery have been obtained by means of a literature review. Qualitative data was collected from 369 participants using focus groups and interviews in 6 European countries (Croatia, Ireland, Portugal, Italy, Spain & The Netherlands). The eHealth Enhanced Chronic Care Model was used as a heuristic tool to integrate the participants’ discourses.

**Discussion:**

The VALUECARE model fits with the features for the integrated care practice facilitating the transformation of care delivery addressing the needs of the growing old population and the rapidly development of technologies.

**Conclusion:**

VALUECARE model highlights best practice value-based, integrated care delivery through the application of a set of 6 guiding principles across 7 different core elements.

## Introduction

Nowadays **people worldwide can expect to live more years**. As consequence, older people (aged 60 years and older) will double (2.1 billion), while the oldest (aged 80 and over) are expected to triple by 2050 (426 million) [1]. This trend started in high-income countries, but it is now also present in low- and middle-income countries. However, and although globally the life expectancy has increased, this has not always been accompanied by good health conditions [2]. In Europe, healthy life years at birth was estimated at 64.5 years for women (that is the 77.6% of their life expectancy) and 63.5 years for men (the 81.9% of their total life expectancy) [3].

As people age, they are more likely to experience different health conditions at the same time [1]; and therefore, the health and social needs of older population grow [4]. This also creates an increasing concern about healthcare systems focused on acute, episodic illness [5]. To respond to this challenge, **integrated care** is presented as a people centred health and care systems which provide life-course quality services adapted to the multidimensional needs of population by a coordinated team of interdisciplinary professionals working at different levels and across different sectors of care in a continuous way [6,7]. However, integrated care is still a complex phenomenon [4]. In this sense, the use of supportive Information and Communication Technology (ICT) has been identified as one of the mechanisms for its successful implementation [8]. However, for this purpose the involvement of end-users in the process of ICT design and implementation as well as iterative flexible process of feedback for ICT adaptations and refining is needed [8]. Thus, the participation of end-users is recognised as the baseline for a successful implementation of supportive digital health and care solutions [9,10,11].

In this context, **co-design** is understood as the method to design better experiences for individuals, their caregivers and care professionals [12]. In fact, the implementation of co-design in healthcare provides great benefits in terms of increased understanding needs and better people’s experiences of care [12,13,14,15]. This can be directly related with the term “value”, used in **value-based healthcare**, as the success degree achieved by a provider in responding the needs of the individuals [16,17], because understanding the patient perspective is integral to delivering high-value, patient-centred care. Value-based healthcare is a current global trend in health and policy defined as the outcomes that matter to the people related to the cost required to achieve these outcomes [18]. In clinical practice, it is reflected in the inclusion of patient-reported outcomes (PROMS), patient-reported experience measures (PREMS) and decision-sharing [19] to involve patients in personalised pathways [20]. Here again, digital technologies can play an important role to measure what matters to the people [21]. The digitalisation of value-based healthcare is only possible if both, the care team, and people trust in this new concept and have confidence in the tools provided. Consequently, a shift in how the healthcare is provided is needed together with professionals training and people’s health and digital literacy [21]. Therefore, value-based healthcare plays a key role to boost fragmentation between health and social care sectors, but also with the community. This is also aligned with the strategic directions of the WHO global strategy on people-centred and integrated health services [22].

In this framework, the purpose of the **VALUECARE model** is to define the way health and social care services are delivered for older adults supported by ICT. The aim of this case report is to get data on the core elements for a value-based, integrated health and social care delivery model which is adaptable to local health and care systems and to the local community assets and supported by ICT solutions. This study presents the construction of the VALUECARE model of care, and the principles integrating the preferences and needs of older adults, care team members – including family and informal caregivers -, health and social service managers and ICT experts. PROMS and PREMS are not considered now, as they will be considered as part of the monitoring and evaluation of the model once it is implemented with the target population at pilot sites level.

## Ethical approval

All participants provided a signed informed consent form as well as their verbal consent prior to participating in the co-design sessions (according to the national regulations in each site). This study was approved by local ethics committees in each pilot site (i.e. the University of Valencia for the activities to be implemented in Valencia city; the Administraçao Regional de Saúde do Centro for the Coimbra (Portugal) pilot site; the University and Clinical research ethics committee in Cork/Kerry (Ireland); the Ethical Committee for Biomedical research of the Faculty of Medicine of the University of Rijeka and the Ethical Committee of Community Health Centre of PrimorjeGorski Kotar Country for the Rijeka (Croatia) pilot site; the Medische Ethische Commissie at Erasmus Medical Center for the Rotterdam (Netherlands) pilot site; and the Comitato Etico per la Sperimentazione Clinica delle Province di Treviso e Belluno in Treviso (Italy)).

## Description of the care practice

### Approach used for the development of the model

A participatory co-design approach was applied to define with the participants a model of care to deliver value-based health and social care supported by ICT solutions. The design was supported by the results of a previous literature review on co-design for people-centred care and digital solutions due to the digital component of the VALUECARE project and care model [9]. This study comprised focus groups and interviews conducted with older adults, health and social care practitioners, informal caregivers, service managers and policy makers. The common inclusion criteria of older people across the 6 pilot sites were: 65 our more years old, confirmed diagnosis of the targeted chronic condition at the time of recruitment, community-dwelling, and able to give consent. In addition, according to the pilot addressed profile an extra inclusion criterion was considered:

- Older people who had a heart attack and finished their rehabilitation in Rijeka (Croatia).
- Older people (75 years or older) with mild to moderate frailty in Cork and Kerry (Ireland) assessed through the Rockwood scale (≤5).
- Older people plus 65 with mild cognitive impairment diagnosis in Treviso (Italy) demonstrated through deficits in neuropsychological tests (resulting in a Clinical Dementia Rating (CDR) score of 0.5), while maintaining complete autonomy in daily living.
- Older people with no or mild cognitive impairment, comorbidities, and lack of social and family support in Coimbra (Portugal).
- Older people who are frail in Valencia (Spain) according to the FRAIL scale (≥1), Barthel index (≥90) and Pfeiffer SMPQ scale (0–2).
- Older people who had a stroke in Rotterdam (The Netherlands).

For informal caregivers, the inclusion criteria were to be responsible of the care of an older person with the above characteristics – depending on the site, be 18 years old or more, and be able to give informed consent. The same for the care team, the unique inclusion criteria were to work with the above target group.

Participants from the consortium countries (Croatia, Ireland, Portugal, Italy, Spain & The Netherlands) were recruited by the local research teams. Each team employed snowball and chain sampling strategies. Finally, a total of 369 participants were involved in the participatory activities, concretely 127 older adults, 109 health and social care practitioners, 86 caregivers, 7 ICT experts, 34 service managers, 3 policy makers and 3 municipality providers.

### Implementation of the participatory approach

**Image 1:**
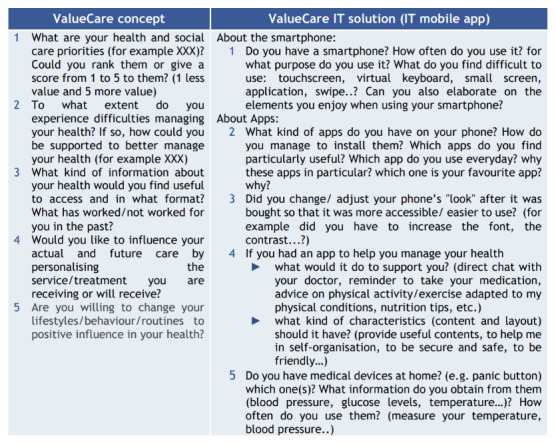
Questions for older people.

**Image 2:**
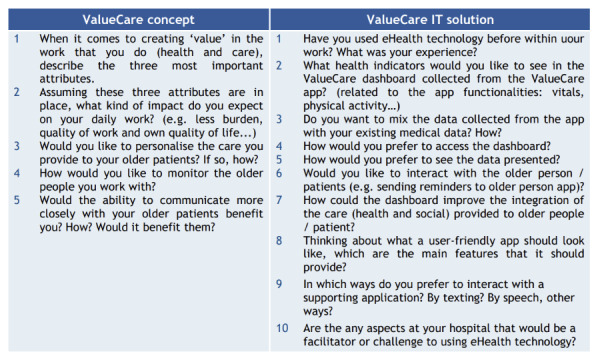
Questions for health and social care practitioners.

**Image 3:**
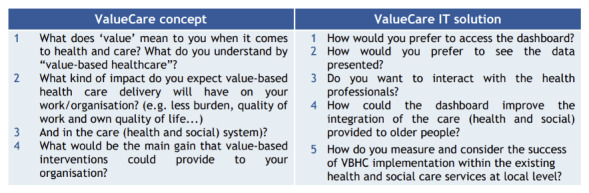
Questions for health and social care managers.

Focus groups and interviews were conducted by local research teams in each of the participating countries with diverse experience in ageing and qualitative research. In concrete a total of 163 interviews (85 interviews with older people, 43 with caregivers, 20 with health professionals, 8 with managers and 7 with ICT experts) and 44 focus groups (6 focus groups with older people, 9 with caregivers, 24 with health professionals, 3 with managers and 2 with policy makers and municipality providers) were implemented across the 6 pilot sites and profiles. All sessions were carried out by the local researchers in their respective national languages combining face-to-face and online means trying to adapt the methodology to the pandemic situation. The common guidelines to perform the co-design activities were shared with pilot sites, consisting of:

- Detailed description and materials to perform focus groups and interviews with the target population.
- Tips to implement those activities online to respond to the pandemic situation and avoiding the potential digital gap of older participants.
- Resources to facilitate participants engagement (project videos for pilot sites to explain the project) and to support the implementation of the suggested activities (templates, checklists, and ethical documents).
- A set of questions to be addressed to each target group in relation to the VALUECARE concept and digital solution. The following images present an overview of the questions addressed to older people (image 1), health and social care practitioners (image 2), and managers (image 3), independently on the format (interview or focus group) implemented [23]. According to the context and target chronic conditions, pilots adapted the questions below.

Feedback gathered from previous questions in the in-depth interviews and focus groups was used to define the concept of value, the treatment pathways and the desired IT support solution, that is, to build the VALUECARE model. Each local team sent transcriptions and main relevant quotes in English. For the analysis of the data, the eHealth Enhanced Chronic Care Model [24] was used as a heuristic tool to integrate the discourses of the different stakeholders involved in the delivery of health and social care for older adults and featuring the guiding principles and the core elements for value-based, integrated health and social care for this group supported by ICT solutions. The eHealth Enhanced Chronic Model builds upon the well-established and tested Chronic Care Model that is shown to improve health outcomes for people with chronic conditions. The original model includes six key components of the health system that encourage high-quality chronic disease care: (1) community resources, (2) health system support, (3) self-management support, (4) delivery system design, (5) decision support, and (6) clinical information systems. The enhanced model incorporates the advances on technology, demonstrating how eHealth tools can be used to increase efficiency of patients’ health self-management. As consequence, the enhanced model includes two additional elements: (1) the eHealth education and (2) the feedback loop; and expands the notion of community to include the (3) online community. These factors have been used to guide and structure the feedback received from participants in components that define the new VALUECARE model due to its emphasis on patient-centred care and self-management.

### Building the VALUECARE model

In the provision of value-based, integrated health, and social care supported by ICT solutions, the study has identified the following two main agents:

1. **Older adults:** A successful transformation in the delivery of care for older adults requires an older adult who is well informed (Rijeka, València, Treviso, Rotterdam, and Cork/Kerry), active (Rijeka, València and Cork/Kerry) and empowered (Treviso, Rijeka), being part of the design and decision-making process of the choices regarding their own wellbeing (València, Rotterdam, Cork/Kerry, and Rijeka).
2. **Interdisciplinary Care team:** This team is understood as the group of health and social care practitioners, family members and informal caregivers who together, analyse, synthesise, and harmonise links between the different stakeholders into a coordinated and coherent whole, and therefore play a significant role in the lives of the older adult. The results of the co-design sessions have stressed the need for creating these interdisciplinary care teams to manage the needs of the individual and support families in the care provided (València, Cork/Kerry, Rijeka, Treviso). The specific requirements for the delivery of value-based, integrated health and social care for older needs are:
  **2.1**. Health and care practitioners: The older adults as well as the family members and informal caregivers stressed the need of the same practitioners of reference (València, Rijeka and Treviso) and a proactive care team (Rotterdam, Rijeka, Cork/Kerry and València). Moreover, practitioners as well as family members and caregivers highlighted the importance for the health and social practitioners to show strong social skills such as empathy (València, Treviso, Rijeka, Cork/Kerry, and Treviso).
  **2.2**. Family and informal caregivers: The participants in the co-design sessions highlighted the need to be prepared for the activity of caring for an older adult, by having self-care/psychological support to prevent burn-out (Rotterdam, Cork/Kerry).

According to the participants’ feedback, the VALUECARE model should consider the following features:

**Continuous and close feedback loop (A):** value-based, integrated health and social care should be based on a trusty relationship between the older person and their care team.

All the co-design sessions referred to the relationship between the older person and their care teams, especially their key worker (social or health professional). In this regard, this continuous and close communication accompanies the individuals in accepting and processing the consequences of ageing and their health status as well as managing their expectations (Rotterdam). Participants identified the following requirements for reinforcing the relationship between the individual and their care team and facilitating a value-based, integrated health and social care model:

1. Older people benefit from having in-person contact with the same key team of active and empathetic practitioners that provide timely, clear answers, information, and guidance (València, Treviso and Rijeka).
2. Family and informal caregivers require an improvement in the communication with the health and care team (Cork/Kerry and Treviso). Furthermore, their capacity to listening, demonstrating strong social skills (València, Treviso, Rijeka and Cork/Kerry) but also the time dedicated to the person they care for (València, Rijeka and Treviso) are key to the care delivery.
3. Practitioners emphasised the importance of good interpersonal relationships and mutual respect (Rijeka and Treviso), together with the recognising professionals’ values of professional expertise and knowledge, humanitarianism, morality, and attentive listening (Rijeka, València and Treviso).
4. Managers also value the communication flow between care teams and the individual (Rijeka, Rotterdam, València, Cork/Kerry, and Treviso), but also among the members of the interdisciplinary care team as this communication allow the care team to manage better the time dedicated to patients (Coimbra), avoid unnecessary visits (València and Cork/Kerry), fostering preventive actions (València and Cork/Kerry), etc. All these can be translated in an efficient health and social system, less consume of resources and better care provision (València, Rijeka and Cork/Kerry).

**e-Health literacy and education (B):** value-based, integrated health and social care should inform and empower older adults to allow them to participate in the decision-making process of their own wellbeing (València, Rotterdam, Cork/Kerry, and Rijeka), as well as highly educated care team members who are knowledgeable of the use of the new technologies in health and care delivery.

For the adoption of a change in care delivery, older persons and care team members require a good level of health literacy, education, and training. The co-design sessions highlighted the need for education and improved skills in e-health. In this regard, the persons’ participation in the decision-making process require that the individuals are informed and empowered to have the capability to decide on personal goals with the guidance of health and social care practitioners (València, Rotterdam, Cork/Kerry, and Rijeka). That is, providing them with accessible information about: (a) their own condition/disease, treatment, and what to expect throughout their condition/disease trajectory (Rijeka, València, Treviso, Rotterdam, and Cork/Kerry); (b) practical guidelines regarding their care pathway (specialists involved in the care pathway, referrals, available services, etc.) (Rijeka, València, and Cork/Kerry); (c) treatment specifications (València, Treviso, and Cork/Kerry); (d) personal health record and their own health related data (València); (e) resources where to find available support (Rijeka, València and Cork/Kerry). Moreover, the care team highlighted some self-identified needs to be addressed to successfully deliver value-based, integrated care for older adults supported by ICT solutions such as education on how to deliver timely, quality evidence-informed care for older adults.

**Comprehensive support and monitoring (C):** value-based, integrated health and social care should provide a continuous and complete support to older adults and their families.

Because of the previous characteristics, the value-based, integrated health and social care supported by ICT solutions will require continuous support and monitoring of the progress of the care plan by the interdisciplinary care team (Coimbra). The care plan should be reviewed and revised in line with the achievement of goals and health status to sustain and hopefully improve older adults’ quality of life over time (Rijeka). Thus, older people demand continuous positive long-term support (Cork/Kerry) not only on their health status but also in the promotion of an active and healthy living, considering the social dimension (Coimbra, València, Cork/Kerry and Rijeka), and focusing more on their wellbeing than on drugs (València). Informal caregivers asked for psychosocial support for their own wellbeing (Coimbra), reduction of bureaucratic tasks (València, Treviso and Rijeka) and in-kind help for the care they provide (Coimbra).

**Health and care decision support (D):** value-based, integrated health and social care should support the participation of older people in the decision making of their own care (València, Rotterdam, Cork/Kerry, and Rijeka).

The co-design sessions with members of the care team also highlighted the need of having access to the health-related information of the older adults as well as their progress in achieving the goals of the care plan (Rijeka, València, Treviso, Rotterdam, and Cork/Kerry). With the aim of providing value-based integrated health and social care for older adults supported by ICT solutions, the information needs to be integrated and shared by the older adult and all the members of their care team to provide a common understanding of the status of each individual and provide coordinated support.

**Care information system/sharing (E):** value-based, integrated health and social care can be supported by a digital solution also co-designed with end-users to leverage the support, monitoring and self-management of older people wellbeing and alleviate the workload of the care team and health/social system providing quality care and contributing to efficient systems. In this sense, the co-design activities reported a Wishlist of the features to be included in the digital solution that supports the value-based integrated care. Although each site provided its own Wishlist according to the different end-users, the following common desired features can be extracted:

- The digital solution should facilitate and support the care provided to the older people and should not entail an extra bureaucratic task for older people/their family or workload for the care team.
- The digital solution should be easy to use according to the digital literacy of older people and friendly to be used by the care team. Participants in the co-design activities stressed the need of training to use any digital solution proposed.
- In addition to its usability, some indications had been highlighted regarding the digital solution for older people:
  - Design: font size, voice activation, use of images rather than text, adaptable to their needs, simple, and safe.
  - Content: reminders, general advice, easy to understand information, motivational messages for healthy and active living, trusty communication channel, and self-care support. In this sense, participants stated the importance of including only meaningful features and information for the older people to not over saturate them.

Regarding the digital solution for professionals there was a common understanding that the solution will contribute to the coordination of health and social care and to the quality of the care provided to older people, allowing professionals to gather information from their patients in an efficient manner and have a complete monitoring of the care proposed.

**Integrated delivery system design (F):** Value-based integrated care delivery requires integration and coordination of appropriate equipment and staff resources within the institution and among different agencies and sectors. Good working conditions with a balanced practitioner-patient ratio allowing quality time with each patient and an optimal use of existing resources. Planning integrated care process pathways for the prevention, monitoring, evaluation, and follow-up, and until the individual is no longer a participant of a service, would facilitate the shift towards a value-based integrated health and care delivery and the information flow.

**Health and care systems change (G):** From the co-design sessions, it is understood that for the implementation of value-based, integrated care models, a paradigm shift is required from a supply-driven to a person-centred demand-driven model, that enables specific health and care service delivery to the individuals’ needs and desires [25] as well as giving older adults a more active role, considering them (and, if it is the case, their caregivers) as active participants in the design of the services and the decision-making process of the care they are offered, receive and involving their community. In this regard, the principles of value-based, integrated health and social care focuses on the values of the individuals and how they would like to be supported, respecting their identity and autonomy with the promotion of self-management activities and support, considering social determinants of health, physical and psychological conditions in the care provided and meeting individuals’ expectations.

Across the key elements presented above, the following guiding **principles** were also incorporated to the VALUECARE Model:

1. **Person Centeredness:** the VALUECARE model requires putting the patient/person at the center of the care design and delivery with continuous and close feedback loop from the care team in relation to their health status and health achievements (A), empowering patient in how to promote their own health (B) and to participate in the design of its own care plan (D) thanks his/her access to their health data (E) and health literacy (B).
2. **Output-based service delivery:** VALUECARE model is based on the active participation of patients in the definition of their own health goals supported by the care team (A, B, C). This active role is directly linked with the health reported benefits that can be also monitored by themselves (C, E) and act as a health motivation tool.
3. **Coordinated health and social care:** the VALUECARE model requires coordinated multidisciplinary care teams to provide patients’ comprehensive support and monitoring (F, C) and best-informed definition of care pathways together with empowered patients (D). For that, a change of traditional care is needed to the VALUECARE person-centered demand-driven model, that enables specific health and care service delivery to the individuals’ needs and desires (G).
4. **Quality and safety:** quality is a recurrent adjective identified when defining the VALUECARE model to provide quality care and contributing to efficient systems. For instance, when highlighting the importance of patients’ education on how to deliver timely and quality evidence-informed care (B) and to monitor the achievement of goals detailed in the personalised care plans to improve older adults’ quality of life over time (C).
5. **Technology as supportive tool:** there is a common understanding that the solution will contribute to the coordination of health and social care and to the quality of the care provided to older people, allowing professionals to gather information from their patients in an efficient manner and have a complete monitoring of the care proposed (E, F).
6. **Sustainability of VALUECARE model:** VALUECARE model is designed according to the needs and preferences of its end-users, this approach is considered to guarantee the sustainability of the model and the effectiveness of the health and care systems because the better use of dedicated resources, that it is possible because the implementation of integrated care (F, G, E, D).

**Figure 1:**
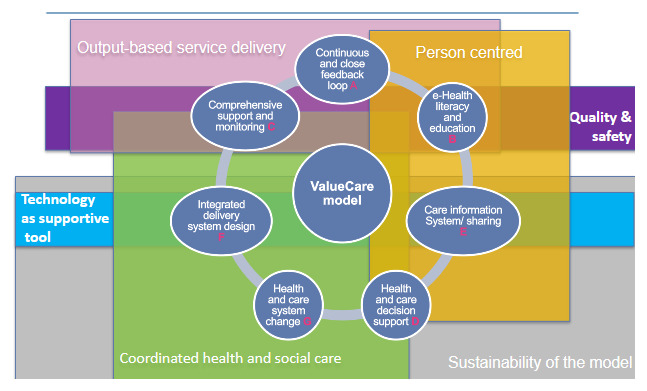
VALUECARE model.

The following figure (Figure 1) represent the main features of the VALUECARE model described above:

## Discussion

The results of the co-design activities reported 6 guiding principles across 7 different core elements for the ValueCare model. The core elements and guiding principles are in line with existing literature on the field, but the results of this study allow partners to build a comprehensive value-based care model to be implemented and tested according to end-users needs. Thus:

Continuity of care and the possibility to choose the care team is associated with patient’s trust [26]. The trust in the care team also influences participants’ involvement in their care path and the sense of decision sharing supported by the care team. That is, patients and informal caregiver demand **continuous and close communication (A)** along their care pathway to build a trusty relationship patient-care team. However, many studies revealed that older people do not enjoy the benefits of e-Health [27]. In this sense, **e-Health literacy and education (B)** tailored to older people needs and profiles is needed together with family support [27] to empower patients in self-management of their health [28] and crucial in making related informed decisions [29]. Care professionals also need education/training to support the successful implementation of e-Health resources [30] and support patients in the sharing decision making. Ensuring scientific and accurate e-Health information and their access [27] – **Health and care decision support (D) –** is critical to allow patients to know their health status and progress and take the best-informed decisions.

Above ValueCare core elements are in line with Nutbeam (2009) [31] that links health literacy with knowledge and skills on the following three levels: (1) functional health literacy that is related with the basic knowledge of the body necessary to understand simple health advices; (2) interactive or communicative health literacy that are communicative skills that helps maintaining own health in interaction with healthcare professionals, that is the **continuous and close communication (A)**; and (3) critical health literacy, that is the ability to critically evaluate the available health information – **Health and care decision support (D)**, that is also related with the corresponding **e-Health literacy and education (B)**.

As the literature reveals, continuous and motivating care **-comprehensive support and monitoring (C) –** is essential to promote healthy behaviors [32]. In this framework, **digital supportive technologies** are promising tools to share health information across providers, settings, and time [33] – **Care information system/sharing (E) –** and can reduce the patient-care burden experienced by health care professionals [34]. This implies the communication between interdisciplinary care teams that requires a high degree of **coordinated health and social care**, both between health professionals, and within treatment levels and settings [32], that is **integrated delivery system design (F)**.

Previous core elements require a a paradigm shift – **Health and care systems change (G) –** from a supply-driven to a person-centred demand-driven model. Health systems need to change to respond to a more holistic and **person Centeredness** approach [35]. This change promises many potential gains, including increased **quality and safety**, improved adherence to care plans, improved treatment and health outcomes, increased patient satisfaction with care, and improved quality of life for patients and their families, the community and society at large [35]. Consequently, enabling **output-based service delivery**, shared between patient and the care team and based on the needs and preferences of its end-users. The benefits also are related with cost-effective and **sustainable health system** because the delivery of appropriate health care, based on end-users needs and preferences and empowered patients, families, and communities.

The VALUECARE model co-designed in this study fits with the features for the integrated practice presented by Porter and Lee (2020) [36] facilitating the adoption of these results into the transformation of care delivery in geriatric care and addressing the needs of the growing old population and the rapidly development of technologies [37].

## Lessons learnt

The participatory design of the VALUECARE model and its results generated the following key learnings:

1. Developing a new model of care requires the active participation of end-users from a holistic point of view, that is, patients, families and the care team, together with ICT developers and managers. This approach fosters the sustainability and acceptability of the proposed innovative solution.
2. Training and empowerment in the new model but also in e-Health are key aspects for patients and the care team to use the solution effectively. Sharing health information with patients is also key to advance in the sharing decision making process.
3. Digital solutions are well received as supportive tools not replacing the human interaction patient-care team.
4. It is needed a paradigm shift from a supply-driven to a person-centred demand-driven model, this change requires training and awareness raising about the benefits and contribution to the sustainability of European healthcare systems.

## Conclusions

VALUECARE model highlights best practice value-based, integrated care delivery through the application of a set of 6 guiding principles across 7 different core elements of the care model, reported by end-users in co-design activities implemented across 6 European sites. Based on the Enhanced Chronic Care Model, these 7 components are continuous and close feedback loop, e-Health literacy and education, comprehensive support and monitoring, health and care decision support, care information system/sharing, integrated delivery system design and health and care systems change. The 6 basic principles for the delivery of health and social care model are person centeredness, output-based service delivery, coordinated health and social care, quality and safety, technology as supportive tool and sustainability of VALUECARE model. The use of the Enhanced Chronic Care Model guided the authors process to build the VALUECARE model, providing a structure to integrate participant’s feedback. The use of an existing model is a powerful tool to support researchers willing to modulate the information gathered in participative activities.

VALUECARE model provides a value-driven care delivery model within health and care systems around the needs of older adults, to ensure that they get the right care, at the right time, by the right team, and in the right place, as they progress through the stages of a condition, injury, or event.

This case report presents some limitations as the results are based on participants from 6 pilot sites around Europe. Further research is needed to validate the principles and core elements in other settings and with older people with other pathologies. The ValueCare is now being tested in 7 pilot sites and will be fine-tuned based on the participants’ feedback, measuring also the outcomes provided by the model in terms of PROMS and PREMS.
